# A decade of Benzodiazepine and Z-drug use in Hong Kong: a longitudinal study

**DOI:** 10.1016/j.lanwpc.2025.101591

**Published:** 2025-06-10

**Authors:** Kyung Jin Lee, Yue Wei, Shek-Ming Leung, Caige Huang, Hei Hang Edmund Yiu, Eunice Kehui Deng, David J. Castle, Simon S.Y. Lui, Vincent K.C. Wong, Ian C.K. Wong, Esther W. Chan

**Affiliations:** a Centre for Safe Medication Practice and Research, Department of Pharmacology and Pharmacy, LKS Faculty of Medicine, The University of Hong Kong, Hong Kong SAR, China; b Laboratory of Data Discovery for Health (D^2^4H), Hong Kong Science Park, Hong Kong Science and Technology Park, Hong Kong SAR, China; c Department of Pharmacology and Pharmacy, LKS Faculty of Medicine, The University of Hong Kong, Hong Kong SAR, China; d Centre for Mental Health Service Innovation, Statewide Mental Health Service, Tasmania, Australia; e University of Tasmania, Hobart, Tasmania, Australia; f Department of Psychiatry, The University of Hong Kong, Hong Kong SAR, China; g Department of Pharmacy, Queen Mary Hospital, Hong Kong SAR, China; h Medical Sciences Division, School of Pharmacy, Macau University of Science and Technology, Taipa, Macau; i Aston School of Pharmacy, Aston University, Birmingham, UK; j The University of Hong Kong Shenzhen Institute of Research and Innovation, Shenzhen, China; k Department of Pharmacy, The University of Hong Kong-Shenzhen Hospital, Shenzhen, China

**Keywords:** Benzodiazepine, Z-drug, Prescribing trend, Long-term use, Electronic health records

## Abstract

**Background:**

Concerns are growing about the long-term use of benzodiazepines (BZDs) and non-benzodiazepines (Z-drugs) due to adverse effects such as drug tolerance, dependence, cognitive dysfunction, and falls, particularly in the elderly. This study aims to understand thorough prescribing patterns of BZDs and Z-drugs across age groups in clinical settings of Hong Kong, especially the long-term prescriptions.

**Methods:**

Using territory-wide electronic health record data from Hong Kong (2014–2023), we analysed the prevalence, incidence, and duration of BZD and Z-drug prescriptions in adults. Long-term use was defined as prescriptions exceeding 90 days. Joinpoint regression models assessed trend changes, focusing on four age groups: 18–25, 26–49, 50–64, and ≥65. Psychiatric diagnoses within 180 days before and after treatment initiation were also evaluated.

**Findings:**

Patients with BZD and Z-drug prescribing increased from 2014 to 2023, with an average annual percentage change (AAPC) of 3.44 [95% CI: 3.26–3.61] in prevalence and 1.51 [0.64–2.45] in incidence. Trends varied by age: the sharpest increases were observed in young adults aged 18–25 (prevalence AAPC: 9.43 [8.36–10.51]; incidence AAPC: 7.56 [6.19–8.89]), whereas the incidence in those aged ≥65 declined after 2019, although it remained the highest. Prevalence of patients with long-term prescribing rose consistently, particularly in young adults (BZD AAPC: 13.43 [11.98–14.62]; Z-drug AAPC: 12.88 [7.85–18.24]). Depression and dementia were the most common psychiatric diagnoses within 180 days before and after treatment initiation.

**Interpretation:**

These findings highlight the need to review long-term prescribing practices and establish clear guidelines for safe BZD and Z-drug use, especially among young adults.

**Funding:**

No funding has been provided for this research.

**Research in context**

**Evidence before this study**

There have been rising concerns about the long-term use of benzodiazepines (BZDs) and non-benzodiazepines (Z-drugs) regarding possible adverse effects, such as drug tolerance and dependence, as well as cognitive dysfunction and falls, notably in the elderly. Strict monitoring of their prescribing in real clinical practice is recommended. On July 10, 2024, we searched PubMed using the terms “Trend∗” AND “Adult”[Mesh] AND (“Sedative”[Mesh] OR “Hypnotic”[Mesh] OR “Anxiolytic”[Mesh] OR “Benzodiazepine”[Mesh] OR “BZD”[Mesh] OR “Z-drug”[Mesh] OR “Nonbenzodiazepine”[Mesh] OR “GABA-A receptor agonist”[Mesh]), limited to articles published from 2000 to the search date. Our search identified 85 studies, with the study cohorts comprising patients across a range of diseases. Since BZDs and Z-drugs are not restricted to specific patient groups, more research targeting the general population is needed to gain a comprehensive understanding of their use in real-world clinical settings. Additionally, existing studies often investigate BZDs or Z-drugs separately and are predominantly conducted in Europe and America. Limited studies reflect recent prescribing trends in Asia, which faces a rapidly aging population and increasing mental health issues. Furthermore, the few existing Asian studies primarily focus on the elderly or segmented age groups, leaving gaps in understanding broader age-specific trends. Detailed age-specific trend analysis is critical, as mental health needs and medication use vary significantly by age, underscoring the need for targeted interventions.

**Added value of this study**

This study represents the first territory-wide trend analysis of BZD and Z-drug prescribing using electronic health record data in clinical settings in Hong Kong. We found that BZD and Z-drug prescribing is still on the rise, which is unlike previous studies in different regions showing a decreasing trend of BZD prescribing. Importantly, we identified a notable and continuous increase among young adults aged 18 to 25 from 2014 to 2023. In this age group, the prevalence of patients with BZD or Z-drug use increased annually by 9.43%, while the incidence grew by 7.56%. Similarly, the prevalence of patients with long-term BZD prescriptions (lasting more than 90 days) rose by 13.43%, and with long-term Z-drug prescriptions increased by 12.88%. Consistent with previous studies, we confirmed that BZD and Z-drug prescribing remains highly prevalent among the older population aged 65 and above, with a decreasing trend in recent years.

**Implications of all the available evidence**

Our trend analysis using joinpoint regression highlights critical implications for reassessing prescribing practices and promoting the safe use of BZDs and Z-drugs in clinical settings. The rising trend in patients with BZD prescribing in Hong Kong suggests established prescribing habits among healthcare providers, underscoring the need to evaluate whether current research on their safety and efficacy is adequately integrated into local health policies and guidelines. The sharp increase in the number of young adults aged 18–25 with prescriptions demands urgent attention to this demographic’s mental health and drug use patterns. This age group faces unique vulnerabilities, including economic pressures, the adverse effects of excessive social media use, and a heightened risk of suicidal ideation. Given these challenges, if the number of young patients requiring hypnotic or anxiolytic prescriptions continues to rise, it is imperative to ensure they receive the most appropriate, evidence-based, and safest treatment options available.

## Introduction

Benzodiazepines (BZDs) are psychotropic medications indicated for various psychiatric conditions, including sleep and anxiety disorders, acute alcohol withdrawal, and epilepsy. They exert calming and sedative effects by modulating GABAergic neurons and reducing brain activity.^1^ BZDs have been widely used as hypnotics and sedatives due to their effectiveness, rapid onset of action, and variety in strength and duration of action. Despite these advantages, there have been longstanding concerns about their potential for misuse, dependence, and withdrawal symptoms.^2^^,^^3^ In response to these concerns, non-benzodiazepines, so-called Z-drugs, were introduced for insomnia management.^4^ The mechanism of Z-drugs is similar to that of BZDs, but Z-drugs bind specifically to the BZ_1_ receptor, a subtype of GABA receptors.^4^ Although initially thought to be less prone to abuse and dependence than BZDs, studies on Z-drugs have reported issues of tolerance and withdrawal effects, as well as being associated with falls, particularly in prolonged use.^1^^,^^5^

In light of this, most country- and disease-specific clinical practice guidelines recommend limiting the prescribing duration and dosage of BZDs and Z-drugs.^6^, ^7^, ^8^ A recent scoping review of guidelines for their use in adults showed that they were recommended for ‘short-term’ use (treatment duration less than four weeks) only when necessary or as an adjunct medication for anxiety, depression, and insomnia.^9^ The 2023 Beers Criteria, developed by the American Geriatrics Society to enhance medication safety for older adults, classifies both BZDs and Z-drugs as potentially inappropriate for this population. It recommends avoiding their use due to risks like cognitive impairment, falls, and hospitalisations, which outweigh the minimal benefits they provide for sleep improvement.^10^ However, real-world clinical use often deviated from these guidelines, with BZDs and Z-drugs frequently prescribed for extended periods, spanning months and even years.^11^^,^^12^ This gap between recommendations and actual prescribing practices underscores the need to better understand real-life usage patterns of these medications to promote adherence to guidelines and ensure safe use. Furthermore, limited studies reflect recent prescribing trends in Asia, which faces a rapidly aging population and increasing mental health issues. Few existing Asian studies focused on the elderly or included segmented age groups.^13^ Detailed age-specific trend analysis is critical because mental health needs and medication use vary by age, highlighting the need for targeted interventions.

We sought to address these issues by employing a comprehensive prescribing database that covers the entire Hong Kong Territory. Hong Kong has a relatively high prevalence of mental health problems, including insomnia and depression,^14^ which often entails the use of BZDs and Z-drugs. Despite BZDs being included in Hong Kong's Dangerous Drugs Ordinance, a measure similar to the Misuse of Drugs Act in the UK, the abuse of two specific BZDs—triazolam and midazolam— persists.^15^ As per the Central Registry of Drug Abuse Report by the Narcotics Division, Security Bureau in Hong Kong, these drugs and one Z-drug, zopiclone, continue to rank among the most abused tranquilizers.^15^ As such, it is fundamental to analyse prescribing trends of BZDs and Z-drugs over time to evaluate adherence to guidelines and regulations. This study aims to achieve this by investigating prescribing data from adults aged 18 and above between 2014 and 2023. Specifically, it seeks to assess the annual trends in the prevalence, incidence, and long-term use of these medications, as well as evaluate the psychiatric diagnoses recorded within 180 days before and after the initiation of BZD or Z-drug use.

## Methods

### Data source

This study utilised health record data from the Clinical Data Analysis and Report System (CDARS), the Hong Kong Hospital Authority (HA)'s electronic clinical database. The HA, which manages all public hospitals and most specialist and general outpatient clinics, offers a broad spectrum of healthcare services, from primary to tertiary, to the Hong Kong population (over 7.3 million people).^16^ CDARS provides anonymised data on patient demographics, diagnoses, prescriptions, inpatient and outpatient visits, emergency department (ED) admission/discharge information, and laboratory test results available for research and auditing since 1993. In CDARS, prescription records are sorted according to the British National Formulary (BNF) classification, and diagnoses are coded as per the International Classification of Diseases, Ninth Revision, Clinical Modification (ICD-9-CM). CDARS has been used for high-quality research regarding medication safety and effectiveness,^17^, ^18^, ^19^ and its validity and accuracy have been reported in previous studies for chronic conditions including substance use disorders (positive predictive value (PPV) 90.7% [95% CI 87.4%–94.0%]), myocardial infarction (85.4% [78.8%–90.6%]), stroke (91.1% [83.2%–96.1%], chronic obstructive pulmonary disease (81.5% [76.1%–86.9%]), asthma (85.0% [80.1%–89.9%]), and interstitial lung disease (79.0% [74.0%–84.0%]).^20^, ^21^, ^22^, ^23^, ^24^

### Study population and treatment period

This study investigated the general population aged 18 years and above who received at least one prescription of BZD or Z-drug from 2014 to 2023 (study period), excluding individuals with incomplete demographic information, such as sex and date of birth. All BZDs and Z-drugs approved in Hong Kong are included in this study (eTable 1). Treatment periods for each individual were generated by merging sequential or overlapped prescriptions. To evaluate prescribing duration, nonsequential prescriptions were also merged if the gap between the end of one prescription and the start of the next was less than 30 days. This approach accounted for the possibility that patients, particularly those with chronic conditions, might be using medication from a stockpile of previous prescriptions.^25^ For subgroup analyses by drug class (BZD and Z-drug) and type, prescription records were individually combined according to the corresponding subgroup using the aforementioned rule. The time difference between the start and end dates of prescriptions determined the duration of the treatment period.

### Long-term prescription

We stratified prescribing duration into five categories: <31 days, 31–90 days, 91–180 days, 181–365 days, and >365 days. A single prescription period lasting more than 90 days was considered long-term based on expert consensus and as this was the most common definition of long-term use in previous literature.^26^ To address inconsistencies in previous research, which often used varied definitions of long-term use,^26^ sensitivity analyses were conducted using alternative thresholds of >180 days and >365 days. For the annual prevalence of patients with long-term use, we included only prescriptions initiated between January 1, 2014, and December 31, 2022, ensuring at least one year of follow-up. We ascribed each long-term prescription to its commencement year, irrespective of its end year.

### Prevalence, incidence, and associated psychiatric diagnoses

The annual prevalence of patients with BZD or Z-drug use was estimated as follows: $\text{annual}\;\text{prevalence}=\frac{Number\;of\;patients\;with\;at\;least\;one\;prescription\;in\;a\;given\;year}{mid-year\;population}*1000$.^27^ Similarly, annual incidence was estimated as: $\text{annual}\;\text{incidence}=\frac{Number\;of\;patients\;with\;an\;incident\;prescription\;in\;a\;given\;year}{mid-year\;population}*1000$. Each patient was counted only once per year, regardless of the number of prescriptions they received within that year. However, patients could be counted in multiple years if they received prescriptions in different years. An *incident prescription* was defined as a new prescription initiated without prior records in the preceding 12 months. Therefore, a patient may have more than one incident prescription during the whole study period if they discontinued the use of BZD or Z-drug for more than a year and then resumed its use. The evaluation of psychiatric diagnoses focused solely on incident BZD and Z-drug prescriptions during the study period. All psychiatric diagnoses made within 180 days before or after the start date of each incident prescription were identified using the ICD-9-CM codes (eTable 2). Consequently, multiple diagnoses could be captured for a single prescription.

### Statistical analysis

Trends in BZD and Z-drug prescribing were analysed using joinpoint regression. This method divides the study period into continuous segments, estimating the Annual Percentage Change (APC) for each segment, which represents the rate of change (increase or decrease) within that specific time interval.^28^ The points where these segments connect, ‘joinpoints,’ indicate statistically significant shifts in the trend. Additionally, this method estimates the Average Annual Percentage Change (AAPC), which summarises the overall trend across the entire study period by calculating a weighted average of the APCs from all segments. The weight for each APC is proportional to the length of its corresponding segment relative to the total study period. While the APC provides detailed insights into the rate of change within specific intervals, the AAPC offers a single, comprehensive measure of the average rate of change over the entire study period. Therefore, in cases without joinpoints, the AAPC equals the APC. The AAPC and APC were estimated on a logarithmic scale, providing a 95% confidence interval (CI).

We identified the number of joinpoints using the grid search method with the weighted Bayesian Information Criterion (BIC). The calendar year served as an independent variable, with assumptions of constant variance and uncorrelated errors. To test these assumptions, we employed the Breusch–Pagan test and the Ljung–Box test, respectively.

Subgroup analyses were conducted by sex, drug classes and types, prescribing durations, and age groups to examine differential trends. Age was calculated as the difference between the prescription start date and the date of birth, divided by 365.25, and categorised into 18–25, 26–49, 50–64, and 65 and above. This age categorisation was designed to reflect significant biological and social transitions that can influence mental health and medication use patterns, with relatively narrower age bands to evaluate the younger age group of 18–25 years, and aligns with previous studies.^29^^,^^30^ In addition to sensitivity analyses for long-term prescribing thresholds (>180 and >365 days), we also divided the study period into pre- and post-COVID phases (2014–2019 and 2020–2023, respectively) to assess pandemic-related changes in trends.

We designed and conducted the study following the STROBE guidelines. Statistical analyses were performed using RStudio version 4.3 (R Group for Statistical Computing) and Joinpoint software version 5.1.0,^31^ considering a p-value < 0.05 as statistically significant. Two investigators (KJL and YW) independently conducted the analyses for quality assurance.

### Ethics approval

This study was approved by the institutional review board of the University of Hong Kong/Hospital Authority Hong Kong West Cluster on April 16, 2024, with an approval number of UW24-211. This study does not require informed consent forms from the participants because anonymised electronic hospital records were utilised without direct contact with patients.

### Role of the funding source

No funding has been provided for this research.

## Results

### Cohort characteristics

The study identified 12,145,825 BZD and Z-drug prescriptions issued to 724,965 patients in Hong Kong from 2014 to 2023 after excluding 311 patients and their prescription records due to incomplete demographic information (Table 1). Of these patients, 71.5% (n = 518,381) were prescribed BZDs, 53.7% (n = 389,296) were prescribed Z-drugs, and 25.2% (n = 182,712) received both BZDs and Z-drugs during the study period. The cohort comprised more women (57.0%, n = 413,153) than men (43.0%, n = 311,812).

**Table 1:** Characteristics of patients who were prescribed BZDs or Z-drugs at least once in 2014–2023. Comorbid conditions were evaluated at or before the start date of the first prescription.

	Overall	BZD**s**^a^	Z-drug**s**	BZD**s** & Z-drug**s**
**No. of prescriptions (%)**	12,145,825	7,626,973 (62.8)	4,518,852 (37.2)	
**No. of patients (%)**	724,965	518,381 (71.5)	389,296 (53.7)	182,712 (25.2)
**Demographics**				
Age at first prescription				
Median in year (Q1, Q3)	60 (46, 74)	59 (45, 72)	62 (49, 76)	58 (45, 71)
Age 18–24, No. (%)	30,864 (4.3)	25,457 (4.9)	12,017 (3.1)	7858 (4.3)
Age 25–49, No. (%)	184,980 (25.5)	142,191 (27.4)	91,390 (23.5)	52,067 (28.5)
Age 50–64, No. (%)	213,042 (29.4)	154,827 (29.9)	113,869 (29.2)	56,905 (31.1)
Age 65 and over, No. (%)	296,079 (40.8)	195,906 (37.8)	172,020 (44.2)	65,882 (36.1)
Sex				
Male (%)	311,812 (43.0)	230,258 (44.4)	153,255 (39.4)	71,701 (39.2)
Female (%)	413,153 (57.0)	288,123 (55.6)	236,041 (60.6)	111,011 (60.8)
**Baseline comorbidities, No. of patients (%)**				
Psychiatric disorder				
Total	267,587 (36.9)	217,010 (41.9)	161,038 (41.4)	90,232 (49.4)
Depression	119,777 (16.5)	98,758 (19.1)	80,048 (20.6)	46,782 (25.6)
Anxiety disorder	36,344 (5.0)	32,540 (6.3)	19,485 (5.0)	12,631 (6.9)
Sleep disorder/disturbance	33,118 (4.6)	24,153 (4.7)	22,580 (5.8)	10,522 (5.8)
Dementia	33,402 (4.6)	23,464 (4.5)	23,031 (5.9)	8207 (4.5)
Schizophrenia	31,888 (4.4)	27,824 (5.4)	18,476 (4.8)	12,933 (7.1)
Other non-organic psychosis	28,185 (3.9)	25,117 (4.9)	16,737 (4.3)	10,857 (5.9)
SUD	27,157 (3.8)	23,472 (4.5)	14,592 (3.8)	9051 (5.0)
Intellectual disability	9715 (1.3)	9137 (1.8)	3129 (0.8)	2421 (1.3)
Bipolar disorder	9234 (1.3)	8408 (1.6)	5902 (1.5)	4282 (2.3)
Personality disorder	6734 (0.9)	6004 (1.2)	4188 (1.1)	2965 (1.6)
OCD	3310 (0.5)	2965 (0.6)	1607 (0.4)	1106 (0.6)
ADHD	1393 (0.2)	1165 (0.2)	554 (0.1)	310 (0.2)
Eating disorder	862 (0.1)	727 (0.1)	483 (0.1)	312 (0.2)
Two diagnoses	46,114 (6.4)	40,359 (7.8)	29,538 (7.6)	29,538 (16.3)
Three diagnoses	10,298 (1.4)	9599 (1.9)	6977 (1.8)	6977 (3.8)
Cardiovascular disease				
Total	172,528 (23.8)	117,050 (22.6)	100,395 (25.8)	38,047 (20.8)
Hyperlipidaemia	84,627 (11.7)	58,383 (11.3)	49,987 (12.8)	17,648 (9.7)
Cerebrovascular disease	71,140 (9.8)	49,809 (9.6)	40,885 (10.5)	15,259 (8.4)
ASCVD	56,726 (7.8)	37,389 (7.2)	35,071 (9.0)	13,079 (7.2)
Congestive heart failure	35,055 (4.8)	22,501 (4.3)	22,550 (5.8)	7530 (4.1)
Myocardial infarction	20,695 (2.9)	13,765 (2.7)	12,899 (3.3)	4209 (2.3)
Peripheral vascular disease	8044 (1.1)	5521 (1.1)	4796 (1.2)	1657 (0.9)
Two diagnoses	27,715 (3.8)	18,542 (3.6)	17,070 (4.4)	6035 (1.6)
Three diagnoses	9653 (1.3)	9653 (1.9)	6325 (1.6)	2091 (1.1)
Cancer				
Total	106,698 (14.7)	68,242 (13.2)	70,741 (18.2)	27,151 (14.9)
Breast	17,950 (2.5)	9627 (1.9)	13,620 (3.5)	4605 (2.5)
Lung	17,678 (2.4)	12,473 (2.4)	12,551 (3.2)	5433 (3.0)
Colorectum	17,297 (2.4)	10,796 (2.1)	11,220 (2.9)	3893 (2.1)
Female genital organ	9423 (1.3)	5929 (1.1)	6482 (1.7)	2628 (1.4)
Lip/Oral/Pharynx	8149 (1.1)	5708 (1.1)	5020 (1.3)	2217 (1.2)
Epilepsy/Seizure	15,778 (2.2)	14,423 (2.8)	5687 (1.5)	3484 (1.9)
Diabetes	98,348 (13.6)	66,826 (12.9)	58,027 (14.9)	22,174 (12.1)

BZD: benzodiazepine, SUD: substance use disorder, OCD: obsessive-compulsive disorder; ADHD: attention deficit hyperactivity disorder, ASCVD: atherosclerotic cardiovascular disease.

a Note: The BZD, Z-drug, and BZD & Z-drug groups are not mutually exclusive; a patient may be included in more than one group.

Prior to or at the time of the first BZD or Z-drug prescription, 36.9% (n = 267,587) of the cohort had a psychiatric diagnosis. Depression was the most common diagnosis (16.5% of the whole cohort (n = 119,777), 19.1% of BZD users (n = 98,758), 20.6% of Z-drug users (n = 80,048), and 25.6% of both BZD & Z-drug users (n = 46,782)), followed by anxiety (5.0%, n = 36,344; 6.3%, n = 32,540; 5.0%, n = 19,485; 6.9%, n = 12,631), sleep disorder/disturbance (4.6%, n = 33,118; 4.7%, n = 24,153; 5.8%, n = 22,580; 5.8%, n = 10,522), dementia (4.6%, n = 33,402; 4.5%, n = 23,464; 5.9%, n = 23,031; 4.5%, n = 8207), and schizophrenia (4.4%, n = 31,888; 5.4%, n = 27,824; 4.8%, n = 18,476; 7.1%, n = 12,933). In terms of other baseline medical comorbidities, 23.8% of the cohort (n = 172,528) had cardiovascular disease (CVD), with hyperlipidaemia being the most prevalent (11.7% of the whole cohort (n = 84,627); 11.3% of BZD users (n = 58,383); 12.8% of Z-drug users (n = 49,987); 9.7% of both BZD & Z-drug users (n = 17,648)), followed by cerebrovascular disease (9.8%, n = 71,140; 9.6%, n = 49,809; 10.5%, n = 40,885; 8.4%, n = 15,259) and atherosclerotic cardiovascular disease (7.8%, n = 56,726; 7.2%, n = 37,389; 9.0%, n = 35,071; 7.2%, n = 13,079). Additionally, 14.7% of the cohort (n = 106,698) had cancer, and 13.6% (n = 98,348) had diabetes (see Table 1).

In the 18–25 age group, depression remained the most common diagnosis, affecting 22.3% of all patients (n = 6884), 23.0% of BZD users (n = 5858), 29.9% of Z-drug users (n = 3597), and 29.4% of both BZD & Z-drug users (n = 2313). This group also showed a higher incidence of intellectual disability diagnoses (8.1%, n = 2495; 9.4%, n = 2388; 4.8%, n = 527; 7.4%, n = 578) compared to the overall study cohort (1.3%, n = 9715; 1.8%, n = 9137; 0.8%, n = 3129; 1.3%, n = 2421) (eTable 3 and Table 1). In addition to psychiatric disorders, epilepsy (7.3%, n = 2251; 8.6%, n = 2180; 2.9%, n = 347; 4.4%, n = 345) was the most common baseline comorbidity in young adults. In contrast, 28.1% of the older adults aged 65 and above (n = 83,051) had baseline psychiatric disorders, primarily dementia (10.9%, n = 32,132) and depression (9.9%, n = 29,422) (eTable 4). This age group was observed to have higher rates of diabetes (23.9%, n = 70,817) and CVD (42.4%, n = 125,531) compared to younger adults.

### Annual prevalence of patients with BZD and Z-drug prescription**s**

Prior to fitting the joinpoint regression analysis, model assumptions such as constant variance and uncorrelated error were confirmed. The Breusch–Pagan test for the constant variance assumption yielded no significant evidence of heteroscedasticity (p-value for overall prevalence = 0.28). For the assumption of uncorrelated errors, we first fit the model assuming no correlation and examined the residuals from this initial model. Using the Ljung–Box test, we confirmed that the residuals exhibited no significant autocorrelation, validating the uncorrelated error model (p-value for overall prevalence = 0.083).

**Fig. 1:**
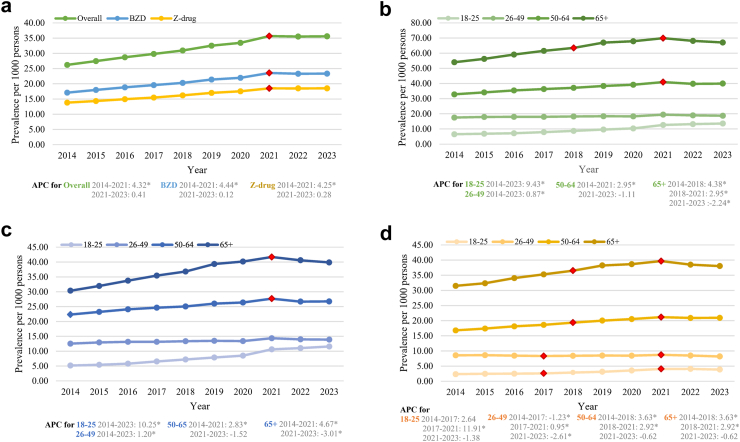
**Prevalence of patients with BZD and Z-drug prescribing in 2014**–**2023**. a) Annual prevalence, which was calculated by dividing the number of patients with prescriptions by the mid-year population, is presented per 1000 persons; b) Overall prevalence by age groups; c) Prevalence of BZD prescriptions by age groups; d) Prevalence of Z-drug prescriptions by age groups. Red points are when the significant trend change happened, which was identified in the joinpoint analysis. An asterisk (∗) indicates that APC (annual percentage change) is significantly different from zero at the alpha = 0.05 level.

Results from joinpoint regression showed that the prevalence of patients with BZD and Z-drug prescribing increased over the study period (AAPC 3.44 [95% CI, 3.26–3.61; p < 0.0001]) (Fig. 1 and Table 2). Similar trends were observed for each drug class (AAPC for BZDs 3.47 [3.23–3.69; p < 0.0001]; AAPC for Z-drugs 3.35 [3.14–3.53; p < 0.0001]). For the trend in different age groups, joinpoint analysis revealed a decline in BZD or Z-drug use among individuals aged 65 and above starting in 2021 (the APC was 4.38 [3.93–5.37; p < 0.0001] from 2014 to 2018, 2.95 [2.41–3.78; p < 0.0001] from 2018 to 2021, and −2.24 [95% CI −3.20 to −1.08; p = 0.0004] from 2021 to 2023). In contrast, prescribing rates among young adults continued to rise (AAPC from 2014 to 2023 9.43 [8.36–10.51; p < 0.0001]) (Fig. 1 and eTable 5).

**Table 2:** Result of joinpoint analyses in prevalence and incidence between 2014 and 2023, and prevalence of long-term prescriptions between 2014 and 2022.

	AAPC [95% CI]	p-value	APC [95% CI]	p-value	Segment
**Prevalence**					
Overall	3.44 [3.26, 3.61]^a^	<0.001	4.32 [4.11, 4.57]	<0.001	2014–2021
			0.41 [−0.45, 1.78]	0.34	2021–2023
BZD	3.47 [3.23, 3.69]	<0.001	4.44 [4.17, 4.77]	<0.001	2014–2021
			0.12 [−1.01, 1.78]	0.72	2021–2023
Z-drug	3.35 [3.14, 3.53]	<0.001	4.25 [4.06, 4.50]	<0.001	2014–2021
			0.28 [−0.78, 1.80]	0.41	2021–2023
**Incidence**					
Overall	1.51 [0.64, 2.45]	<0.001	2.91 [2.18, 5.27]	0.002	2014–2021
			−3.27 [−7.39, 1.00]	0.17	2021–2023
BZD	2.31 [1.49, 3.13]	<0.001	4.11 [3.36, 5.61]	<0.001	2014–2021
			−3.76 [−7.54, 0.89]	0.13	2021–2023
Z-drug	−0.09 [−0.60, 0.44]	0.73	1.77 [0.74, 4.19]	<0.001	2014–2019
			−2.37 [−5.17, −0.96]	<0.001	2019–2023
**Prevalence of long-term prescriptions**					
BZD	1.77 [0.92, 2.65]	<0.001			2014–2022
Z-drug	2.29 [1.92, 2.71]	<0.001	3.26 [2.84, 4.07]	<0.001	2014–2020
			−0.55 [−2.19, 1.56]	0.70	2020–2022

BZD: benzodiazepine, AAPC: average annual percentage change during the whole study period, APC: annual percentage change during the corresponding segment.

a Interpretation: For the overall prevalence, the AAPC is 3.44, indicating an average annual increase of 3.44% from 2014 to 2023. A significant change in trend was identified in the year 2021, which divides the study period into two segments. The **first segment, labelled 2014**–**2021**, covers the period **from the start of 2014 up to the end of 2020**, while the **second segment, labelled 2021**–**2023**, covers the period **from the start of 2021 through the end of 2023**. The APC values are specific to each segment. In the first segment, the APC is 4.32%, indicating an annual increase of 4.32% in prevalence. In the second segment, the APC decreases to 0.41%, indicating a slower annual increase of 0.41% during this period.

Among individual drugs, zopiclone was the most widely prescribed (AAPC 2.98 [2.87–3.09; p < 0.0001], despite a slight decline after 2021 (APC from 2021 to 2023 −0.72 [−1.26 to −0.25; p = 0.0004] (eFig. 1 and eTable 6). Lorazepam, diazepam, and zolpidem were also highly prevalent throughout the study period. Midazolam showed the most substantial increase, particularly between 2014 and 2019 (APC in prevalence from 2014 to 2019 25.63 [21.45–31.01; p < 0.0001], from 2019 to 2023 2.38 [−3.51–7.20; p = 0.33]). Similar results were observed for patients with incident midazolam prescriptions (APC from 2014 to 2019 27.24 [22.28–33.73; p < 0.0001], from 2019 to 2023 2.04 [−5.35–7.51; p = 0.47]).

### Incident cases and common psychiatric diagnoses

**Fig. 2:**
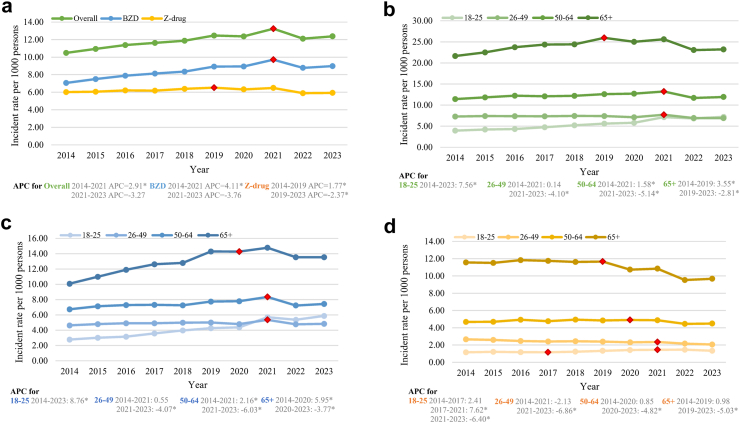
**Incidence of patients with BZD and Z-drug prescribing in 2014**–**2023.** a) Annual incident rate, which was calculated by dividing the number of patients with incident prescriptions by the mid-year population, is presented per 1000 persons; b) Overall incident rate by age groups; c) Incident rate of BZD prescriptions by age groups; d) Incident rate of Z-drug prescriptions by age groups. Incident prescription is defined as new prescriptions without the previous records of the past 12 months. Red points are when the significant trend change happened, which was identified in the joinpoint analysis. An asterisk (∗) indicates that APC (annual percentage change) is significantly different from zero at the alpha = 0.05 level.

The trend of the incidence of patients with new BZD and Z-drug prescribing is shown in Fig. 2. The overall incidence increased from 2014 to 2023 (AAPC 1.51 [0.64–2.45; p < 0.0001]), driven by a rise in BZDs (AAPC 2.31 [1.49–3.13; p < 0.0001]) while the incidence for Z-drugs decreased slightly (AAPC −0.09 [−0.60–0.44; p = 0.73]) (Table 2). Joinpoint regression analysis revealed a decline in incident prescribing after 2021 for overall and BZDs and after 2019 for Z-drugs. Age-stratified analysis of incident cases mirrored the trends observed in prevalent cases (Fig. 2 and eTable 7). While the most significant increase was among individuals aged 18–25, the highest incidence was found in patients aged 65 and older (Fig. 2 and eTable 7). Notably, the incidence of patients with new Z-drug prescribing decreased in recent years across all age groups (after 2021 for groups aged 18–25 and 26–49, after 2020 for the group aged 50–64, and after 2019 for the group aged 65 and above). In contrast, incident BZD prescribing for young adults continuously increased during the study period (AAPC 8.76 [7.28–10.15; p < 0.0001]). In the investigation of the common psychiatric diagnoses among patients with incident prescriptions each year, depression was the most common across all years, followed by dementia and schizophrenia (eTable 8). In comparison with older adults, the most common diagnoses in young adults were schizophrenia, anxiety disorder, substance use disorder (SUD), bipolar disorder, and intellectual disability (eTable 8). In addition, a higher proportion of young adults had multiple psychiatric diagnoses than the older population.

### Prevalence of patients with long-term prescribing

**Fig. 3:**
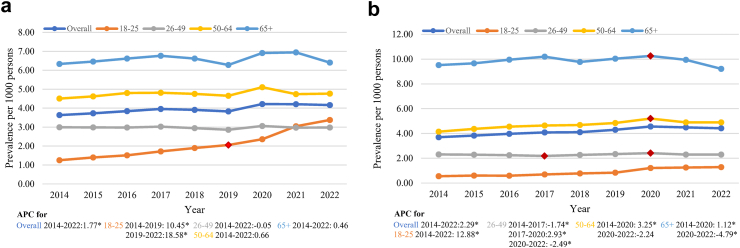
**Annual prevalence of patients with long-term prescriptions (prescribing duration exceeding 90 days).** a) long-term BZD use by age groups; b) long-term Z-drug use by age groups. Prescriptions were followed from the start date with at least one year of follow-up time; hence, prescriptions between January 1st, 2014 and December 31st, 2022 were considered in this analysis. Red points are when the significant trend change happened, which was identified in the joinpoint analysis. An asterisk (∗) indicates that APC (annual percentage change) is significantly different from zero at the alpha = 0.05 level.

During the study period, 44.9% of BZD prescriptions (n = 325,223) and 62.4% Z-drug prescriptions (n = 385,976) exceeded the recommended 30-day usage duration (eTable 9). The prevalence of patients with long-term Z-drug prescriptions (>90 days) increased at a higher rate (AAPC 2.29 [1.92–2.71; p < 0.0001]) compared to long-term BZD prescriptions (AAPC 1.77 [0.92–2.65; p < 0.0001]) (Fig. 3 and Table 2). However, the trend for long-term Z-drug prescriptions showed a slight decrease after 2020 (APC from 2014 to 2020 3.26 [2.84–4.07; p < 0.001], from 2020 to 2022 −0.55 [−2.19–1.56; p = 0.70]).

Age-specific differences were evident in long-term prescription trends, with young adults showing the most significant increases for both BZDs (AAPC 13.43 [11.98–14.62; p < 0.0001]) and Z-drugs (AAPC 12.88 [7.85–18.24; p < 0.0001]). Remarkably, this age group exhibited a steeper increase in long-term BZD prescriptions in recent years (APC from 2014 to 2019 10.45 [4.82–12.60; p < 0.0001], from 2019 to 2022 18.58 [14.00–25.44; p < 0.0001]) while long-term Z-drug prescribing trends did not show significant trend change point (see Fig. 3 and eTable 10).

Subgroup analysis stratified by prescription durations (<31 days, 31–90 days, 91–180 days, 180–365 days, and >365 days) revealed varying trends for Z-drugs (eFig. 2 and eTable 11). Specifically, patients for 91–180 days showed a continuous decline (AAPC −1.67 [95% CI −3.33–0.02; p = 0.054]), while longer durations (181–365 days and >365 days) exhibited a consistent increase, with AAPCs of 2.94 [2.17–3.88; p < 0.0001] and 4.57 [3.95–5.26; p < 0.0001], respectively.

Sensitivity analyses defining long-term use as prescriptions exceeding 180 days and 365 days confirmed increasing trends consistent with the main findings. For BZDs, AAPCs were 3.36 [2.65–4.09; p < 0.0001] for >180 days and 3.70 [2.91–4.49; p < 0.0001] for >365 days. For Z-drugs, AAPCs were 3.85 [3.35–4.28; p < 0.0001] for >180 days and 4.57 [3.95–5.26; p < 0.0001] for >365 days (eFig. 3). Another sensitivity analysis, dividing the study period into pre- and post-COVID phases, revealed that during the pre-COVID period, the prevalence and incidence of patients using BZDs or Z-drugs increased significantly (eTable 12). In contrast, during the post-COVID period, prevalence increased while incidence declined numerically; however, neither change was statistically significant (Overall prevalence 1.84 [−1.34–5.10; p = 0.26]; Overall incidence −0.88 [−5.72–4.08; p = 0.68]) (eTable 12).

## Discussion

This study provides a detailed evaluation of prescribing patterns for BZDs and Z-drugs in the general adult population of Hong Kong between 2014 and 2023. To the best of our knowledge, this is the first territory-wide study leveraging electronic health record data to investigate BZD and Z-drug prescribing in clinical settings in Hong Kong. Our findings highlight three key observations. First, BZD and Z-drug prescribing is prevalent, particularly among the elderly, with a notable recent increase among young adults. Second, long-term prescribing of BZDs and Z-drugs (period exceeding 90 days) is on the rise, with this trend more pronounced for Z-drugs than for BZDs, particularly among young adults. Lastly, significant trend shifts in the prevalence and incidence of prescribing occurred during the study period, with joinpoints varying by drug class (BZD or Z-drug) and across age groups.

Both the prevalence and incidence of patients with BZD prescribing show an upward trend similar to those of Z-drugs. The increasing trend of BZD use contrasts with findings from previous studies in other countries, which reported decreasing usage of BZDs.^32^^,^^33^ This discrepancy might be attributed to factors specific to Hong Kong. For example, BZDs might be more affordable than Z-drugs or local practitioners may have established prescribing habits favouring BZDs. Further research is needed to explore the underlying reasons for this increase in BZD prescriptions. Additionally, the rising trend of BZD and Z-drug prescriptions underscores the need to reassess whether current research on their safety and effectiveness is adequately reflected in local health policies and guidelines for healthcare providers.

Joinpoint analysis identified the year 2021 as a significant change point, showing decreased incidence and a slower increase in prevalence during 2021–2023 compared to 2014–2021. Sensitivity analysis was conducted, which divided the study period into pre- and post-COVID phases based on 2020, revealing consistent trend changes, although these trends lacked statistical significance. The 2021 joinpoint, later than the 2020 cut-off, likely reflects a lagged pandemic effect, where immediate 2020 disruptions evolved into sustained prescribing changes detectable by 2021.^34^

Our findings indicate age-related differences in the prevalence and incidence of patients with BZD and Z-drug prescribing, including long-term prescriptions that lasted over 90 days. Notably, there were sharp increases in young adults aged 18–25. This trend may reflect the growing mental health burden in this population^35^ as well as the reduced stigma associated with mental health issues, leading to a greater willingness to seek and accept treatment. However, the increasing use of these drugs in young adults is of concern, given the association between BZD use and drug overdose risk.^36^ While prior research has focused on the safety of BZD and Z-drug use in elderly patients, our findings emphasise the necessity for greater attention to younger populations. Young adults face unique vulnerabilities, including economic pressures, societal stress to succeed, and the adverse effects of excessive social media use, all of which can be detrimental to their mental health.^35^ Should the number of young patients requiring hypnotic or anxiolytic prescriptions increase, it is crucial to ensure that they receive the most appropriate and safest treatment options.

The highest prevalence and incidence of BZD and Z-drug use were observed among older adults, consistent with findings from previous studies in Western^37^ and Asian countries.^38^ This is concerning given the safety risks, particularly falls, associated with these medications in older populations.^39^, ^40^, ^41^ We did, however, observe a declining trend in the number of both prevalent and incident older patients in recent years, specifically after 2021 for the prevalence and 2019 for the incidence. This perhaps reflects the increased awareness of such risks in this population, potentially prompting a switch to alternative treatments such as melatonin.^5^^,^^42^

Our study also evaluated prescribing duration to assess adherence to guidelines in real-world clinical settings, which recommend durations of less than 30 days. Even with a more lenient definition of long-term use as prescriptions exceeding 90 days, we identified an increasing trend in long-term prescribing, with this trend being more pronounced among Z-drug users. This higher rate of long-term Z-drug prescriptions aligns with findings from other studies despite the varying periods defining long-term use.^43^ The sensitivity analyses assessing trends over extended durations (e.g., >180 days and >365 days) confirmed an upward trend, ensuring the robustness of our findings. Notably, the magnitude of the increase was more pronounced with the longer duration threshold, suggesting the growing prevalence of patients with chronic BZD and Z-drug use. Further research should investigate dose changes over extended prescribing periods and the external factors contributing to long-term prescribing, such as extended waiting times for psychiatric consultations in Hong Kong.

Although not previously reported, we found that the number of patients with long-term Z-drug prescriptions has been increasing at a steeper rate than that with BZDs. One possible explanation for this could be the perception that Z-drugs are safer than BZDs, leading clinicians to prescribe them for more extended periods. Additionally, since Z-drugs are primarily indicated for insomnia, this trend may reflect a rise in chronic insomnia and the associated demand for prolonged treatment. Despite clinical guidelines recommending cognitive behavioural therapy (CBT) as the first-line treatment for insomnia, its accessibility is limited.^43^ In any event, further evidence-based research is needed to compare available therapeutic strategies for insomnia, particularly to determine the most effective approaches for specific patient populations, such as the elderly.

In our study, the most common psychiatric diagnosis in patients with incident BZD or Z-drug prescriptions was depression, followed by dementia, schizophrenia, anxiety disorder, and SUD. This finding aligns partially with previous research focused on comorbidities.^42^ Each of these conditions presents safety risks that must be carefully weighed when prescribing these medications. For example, in patients with depression, especially those at risk of self-harm and suicide, BZDs and Z-drugs can worsen mood symptoms.^44^ Older patients with dementia are at a higher risk of falls and mortality when using these drugs and may also experience exacerbated cognitive impairment.^2^^,^^45^ For patients with SUD or a history of drug misuse, the potential for abuse of BZDs and Z-drugs necessitates their restriction to acute or emergency situations. Further research should be conducted with an in-depth investigation into how BZDs and Z-drugs are prescribed and monitored in terms of their duration and dosage in these vulnerable patient groups.

There are several limitations to our study. First, the CDARS data originated from the public healthcare system, and as a result, prescriptions from private clinics were not included. This could potentially lead to an underestimation of the prevalence and incidence of patients with BZD and Z-drug prescriptions. However, public healthcare institutions primarily provide mental health services in Hong Kong, making our study likely representative of prescribing patterns.^46^ The second limitation is the assumption that prescription records infer medication use. Third, our long-term prescription study did not consider ‘as-needed’ (PRN) prescriptions or the frequency of medication use during the prescription period, which may impact our estimation of long-term trends. Fourth, while there were notable changes in the trend identified between 2014 and 2023, this study primarily aimed to provide a comprehensive overview of the prescribing patterns of BZDs and Z-drugs. Consequently, it did not explore underlying drivers of changes in these trends, such as healthcare policies or external influences, including media reports. Future research should investigate these factors to understand the mechanisms behind the observed trends, enabling more effective interventions to reduce potentially inappropriate use of these drugs. Lastly, the generalisability of our study findings may be limited due to age distribution and the representativeness of the study population, which is predominantly Asian.

Nevertheless, the results of our study still hold value, contributing to the pool of comparative data for future research on how these trends may vary across different nations and healthcare systems. It should also be noted that the large sample size in our study increases the statistical power of the joinpoint regression analysis, enabling the detection of even small changes or subtle trend variations.^47^

## Contributors

Conception and design: KJL, DC, and EWC; Acquisition of data: KJL; Access to raw data: KJL and YW; Verified the data: KJL and YW; Interpretation of data: KJL, YW, and EWC; Statistical analysis: KJL and YW; Drafting the manuscript: KJL; Critical revision of the manuscript for important intellectual content: YW, SML, CH, HHEY, EKD, DJC, SSYL, VKCW, ICKW, EWC; Administrative, technical, or material support: EWC; Supervision: EWC and SSYL. All authors reviewed the manuscript and approved the final version to submit for publication.

## Data sharing statement

Data will not be available to share with others as the data custodians have not given permission.

## Editor note

The Lancet Group takes a neutral position with respect to territorial claims in published maps and institutional affiliations.

## Declaration of interests

ICKW reports research funding from the Hong Kong Research Grants Council, the Hong Kong Health and Medical Research Fund of Health Bureau of the Hong Kong SAR Government, the European Commission, IQVIA and Amgen outside the submitted work; and is a director of Jacobson Medical, Advanced Data Analytics for Medical Science and OCUS Innovation in Hong Kong and a former director of Therakind Ltd in London and Asia Medicine Regulatory Affairs (AMERA) Services Limited in Hong Kong, he was a consultant to IQVIA and World Health Organization; and serves as a member of the Pharmacy and Poisons Board, Hong Kong SAR Government, served expert witness for the Hong Kong Appeal Court; EWC has received grants from Research Grants Council of the Hong Kong SAR, Research Fund Secretariat of the Health Bureau of the Hong Kong SAR, National Natural Science Fund of China, Bayer, Pfizer, Novartis, AstraZeneca, the RGA Reinsurance Company, Narcotics Division of the Security Bureau of the Hong Kong SAR, and the National Health and Medical Research Council Australia; consulting fees from AstraZeneca, Pfizer and Novartis; and honorarium from the Hospital Authority of the Hong Kong outside the submitted work; DJC has received grants from NHMRC and ARC (Australia), Brain Canada (Canada), Milken Institute, and Psyche Foundation; royalties from Cambridge University Press and Oxford University Press; consulting fees from Seqirus, Boehringer Ingelheim, Lundbeck and Servier; honoraria from Servier, Mindcafe, Seqirus, Boehringer Ingelheim, Lundbeck; has financial interests in Optimal Health Program and Clarity Healthcare; is a board member of Clarity Healthcare and an advisor to Tryptamine Therapeutics; HHEY reported receiving research grants from the Health Bureau of the Government of the Hong Kong SAR (HMRF) and Viatris, and an honorarium from the 12th Southern Pharmacoeconomics Forum, outside the submitted work. No other disclosures were reported.

## Appendix ASupplementary data

eFigs. 1–3 and eTables 1–12
