# Supplementary material for: A decade of Benzodiazepine and Z-drug use in Hong Kong: a longitudinal study
Source: Lancet Reg Health West Pac. 2025 Jun 10;59:101591. doi: 10.1016/j.lanwpc.2025.101591 (PMC12178920; doi:10.1016/j.lanwpc.2025.101591)
Supplement: eFigs. 1–3 and eTables 1–12 [file mmc1.docx]

eTable 1. List of all BZDs and Z-drugs included in the study. BZDs are classified as short-acting and long-acting according to their half-life values. Short-acting BZDs have 5-24 hours of half-life, while long-acting ones have a half-life exceeding 24 hours^1^.

| **Drug name** | **Type** | **ACT** | **Action** | **Possible Indication^2^** |
| --- | --- | --- | --- | --- |
| Alprazolam | BZD | Anxiolytic | Short | Anxiety, panic |
| Bromazepam | BZD | Anxiolytic | Short | Anxiety, insomnia |
| Clobazam | BZD | Anxiolytic | Long | Anxiety, epilepsy |
| Clonazepam | BZD | Antiepileptic | Long | Anxiety, panic, seizure |
| Chlordiazepoxide | BZD | Anxiolytic | Long | Anxiety, perioperative sedation |
| Diazepam | BZD | Anxiolytic | Long | anxiety, seizure, perioperative sedation |
| Flunitrazepam | BZD | Hypnotic | Long | anxiety, insomnia |
| Lorazepam | BZD | Anxiolytic | Short | Anxiety, perioperative sedation |
| Midazolam | BZD | Hypnotic | Short | Perioperative sedation |
| Nitrazepam | BZD | Hypnotic | Long | Insomnia, seizure |
| Triazolam | BZD | Hypnotic | Short | Insomnia |
| Zolpidem | Z-drug | Hypnotic | Short | Insomnia |
| Zopiclone | Z-drug | Hypnotic | Short | Insomnia |

*BZD: benzodiazepine, ACT: anatomical therapeutic chemical

eTable 2. ICD9 codes to identify comorbidities and common psychiatric diagnoses of BZD and Z-drug prescribing.

| **Medical Conditions** | **ICD-9-Codes** |
| --- | --- |
| **Mental Disorders** |  |
| ADHD | 314.01 |
| Anxiety disorder | 300.00-300.29 |
| Bipolar disorder | 296.00-296.10, 296.40-296.89 |
| Dementia | 290, 294.1, 294.8 |
| Depression | 296.20-296.36, 300.4, 309, 311 |
| Eating disorder | 307.1, 307.50-307.59 |
| Intellectual disability | 317-319 |
| Personality disorder | 301.0-301.9 |
| OCD | 300.3 |
| Schizophrenia | 295 |
| Sleep disorder/disturbance | 307.4, 780.5 |
| SUD | 303-305 |
| Other psychosis | 298 |
| **Circulatory System** |  |
| ASCVD | 410-414, 430-438, 440-443, |
| Cerebrovascular disease | 430-438 |
| Congestive heart failure | 398.91, 402.01, 402.11, 402.91, 404.01-03, 404.11-13, 404.91-93, 428 |
| Hyperlipidemia | 272.0-4 |
| Myocardial infarction | 410 |
| Peripheral vascular disease | 441, 443.9, 785.4, V43.4 |
| **Cancer** |  |
| Stomach | 151, 230.2 |
| Lip, oral cavity, pharynx | 140-149, 230.0 |
| Colorectum | 153-154, 230.3-4 |
| Liver | 155, 230.8 |
| Pancreas | 157 |
| Lung | 162, 232.0-2 |
| Bone, skin, mesothelial and soft tissue | 170-173, 176 |
| Breast | 174-175, 233.0 |
| Female genital organ | 179, 180-184, 233.1-3 |
| Male genital organ | 185-187, 233.4-6 |
| Bladder | 188, 233.7-9 |
| Kidney | 189 |
| Brain, nervous system | 191-192 |
| Lymphoid and hematopoietic tissue | 280-289 |
| **Epilepsy** | 345 |
| **Diabetes** | 250 |

*BZD: benzodiazepine, ID: intellectual disability, OCD: obsessive-compulsive disorder, SUD: substance use disorder, ASCVD: atherosclerotic cardiovascular disease, ICD: international classification of disease

eTable 3. Baseline comorbidities of young adults aged 18 to 25 at the time or before the first prescription start date.

|  |  | **Overall** | **(%)** | **BZD** | **(%)** | **Z-drug** | **(%)** | **BZD & Z-drug** | **(%)** |
| --- | --- | --- | --- | --- | --- | --- | --- | --- | --- |
| **Total Patient Number** |  | 30,864 |  | 25,457 | (82.5) | 12,017 | (38.9) | 7,858 | (25.5) |
| **Psychiatric**  **Disorder** | TOTAL | 16,230 | (52.6) | 14,208 | (55.8) | 7035 | (58.5) | 4,982 | (63.4) |
|  | Depression | 6,884 | (22.3) | 5,858 | (23.0) | 3,597 | (29.9) | 2,313 | (29.4) |
|  | Other psychosis | 2,906 | (9.4) | 2,712 | (10.7) | 1,466 | (12.2) | 1,233 | (15.7) |
|  | Intellectual disability | 2,495 | (8.1) | 2,388 | (9.4) | 527 | (4.8) | 578 | (7.4) |
|  | Anxiety disorder | 1,596 | (5.2) | 1,458 | (5.7) | 580 | (4.8) | 391 | (5.0) |
|  | Schizophrenia | 1,443 | (4.7) | 1,356 | (5.3) | 761 | (6.3) | 622 | (7.9) |
|  | Sleep disorder/disturbance | 1,179 | (3.8) | 869 | (3.4) | 646 | (5.4) | 308 | (3.9) |
|  | ADHD | 1,086 | (3.5) | 906 | (3.6) | 404 | (3.4) | 238 | (3.0) |
|  | SUD | 1,040 | (3.4) | 943 | (3.7) | 447 | (3.7) | 351 | (4.5) |
|  | Bipolar disorder | 889 | (2.9) | 834 | (3.3) | 527 | (4.4) | 389 | (5.0) |
|  | Personality disorder | 728 | (2.4) | 661 | (2.6) | 390 | (3.3) | 304 | (3.9) |
|  | OCD | 547 | (1.8) | 489 | (1.9) | 216 | (1.8) | 163 | (2.1) |
|  | Eating disorder | 306 | (1.0) | 262 | (1.0) | 123 | (1.0) | 87 | (1.1) |
|  | Dementia | 7 | (0.0) | 6 | (0.0) | 4 | (0.0) | 3 | (0.0) |
|  | Parkinson’s disease | 6 | (0.0) | 6 | (0.0) | 2 | (0.0) | 2 | (0.0) |
| **Epilepsy** |  | 2,251 | (7.3) | 2,180 | (8.6) | 347 | (2.9) | 345 | (4.4) |
| **Diabetes** |  | 195 | (0.6) | 162 | (0.6) | 83 | (0.7) | 51 | (0.6) |
| **Cardiovascular Disease** | TOTAL | 633 | (2.1) | 542 | (2.2) | 184 | (1.5) | 102 | (1.3) |
|  | Cerebrovascular disease | 251 | (0.8) | 225 | (0.9) | 56 | (0.5) | 34 | (0.4) |
|  | Hyperlipidaemia | 120 | (0.4) | 101 | (0.4) | 47 | (0.4) | 24 | (0.3) |
|  | Congestive Heart Failure | 100 | (0.3) | 84 | (0.3) | 38 | (0.3) | 20 | (0.3) |
|  | ASCVD | 38 | (0.1) | 32 | (0.1) | 16 | (0.1) | 7 | (0.1) |
|  | Peripheral vascular disease | 28 | (0.1) | 25 | (0.1) | 10 | (0.1) | 7 | (0.1) |
|  | Myocardial infarction | 7 | (0.0) | 4 | (0.0) | 3 | (0.0) | 0 | (0.0) |
| **Cancer** | TOTAL | 470 | (1.5) | 360 | (16.5) | 230 | (1.9) | 135 | (1.7) |
|  | Bone/skin/mesothelial | 55 | (0.2) | 43 | (0.2) | 36 | (0.3) | 22 | (0.3) |
|  | Brain/CNS | 50 | (0.2) | 50 | (0.2) | 16 | (0.1) | 15 | (0.2) |
|  | Lip/Oral/Pharynx | 33 | (0.1) | 24 | (0.1) | 18 | (0.2) | 11 | (0.1) |
|  | Lymphoid | 32 | (0.1) | 20 | (0.1) | 18 | (0.2) | 8 | (0.1) |
|  | Female genital organ | 31 | (0.1) | 17 | (0.1) | 22 | (0.2) | 7 | (0.1) |
|  | Male genital organ | 20 | (0.1) | 19 | (0.1) | 5 | (0.0) | 3 | (0.0) |
|  | Colorectum | 17 | (0.1) | 10 | (0.0) | 12 | (0.1) | 8 | (0.1) |
|  | Breast | 14 | (0.1) | 11 | (0.0) | 7 | (0.1) | 7 | ((0.1) |
|  | Lung | 7 | (0.0) | 6 | (0.0) | 4 | (0.0) | 2 | (0.0) |
|  | Kidney | 7 | (0.0) | 5 | (0.0) | 4 | (0.0) | 2 | (0.0) |
|  | Liver | 6 | (0.0) | 6 | (0.0) | 1 | (0.0) | 2 | (0.0) |
|  | Stomach | 6 | (0.0) | 4 | (0.0) | 3 | (0.0) | 1 | (0.0) |
|  | Bladder | 1 | (0.0) | 1 | (0.0) | 0 | (0.0) | 0 | (0.0) |
|  | Pancreas | 1 | (0.0) | 1 | (0.0) | 0 | (0.0) | 0 | (0.0) |

*BZD: benzodiazepine, SUD: substance use disorder, OCD: obsessive-compulsive disorder, ADHD: attention deficit hyperactivity disorder, ASCVD: atherosclerotic cardiovascular disease, CNS: central nervous system.

**Note: The BZD, Z-drug, and BZD&Z-drug groups are not mutually exclusive; a patient may be included in more than one group.

eTable 4. Baseline comorbidities of older adults aged 65 and above at the time of or before the first prescription start date.

|  |  | **Overall** | **(%)** | **BZD** | **(%)** | **Z-drug** | **(%)** | **BZD & Z-drug** | **(%)** |
| --- | --- | --- | --- | --- | --- | --- | --- | --- | --- |
| **Total Patient Number** |  | 296,079 |  | 162,088 | (54.7) | 133,991 | (45.3) | 65,882 | (22.3) |
| **Psychiatric Disorder** | TOTAL | 83,051 | (28.1) | 47,311 | (16.0) | 35,740 | (12.1) | 25,857 | (39.2) |
|  | Dementia | 32,132 | (10.9) | 15,879 | (5.4) | 16,253 | (5.5) | 7,810 | (11.9) |
|  | Depression | 29,422 | (9.9) | 17,070 | (5.7) | 12,352 | (4.2) | 10,471 | (15.9) |
|  | Sleep disorder/disturbance | 12,671 | (4.3) | 6,476 | (2.2) | 6,195 | (2.1) | 3,558 | (5.4) |
|  | Anxiety disorder | 11,347 | (3.8) | 8,060 | (2.7) | 3,287 | (1.1) | 3,998 | (6.1) |
|  | Other psychosis | 6,912 | (2.3) | 4,144 | (1.4) | 2,768 | (0.9) | 2,009 | (3.0) |
|  | SUD | 6,438 | (2.2) | 4,426 | (1.5) | 2,012 | (0.7) | 1,595 | (2.4) |
|  | Parkinson’s disease | 6,046 | (2.0) | 3,757 | (1.3) | 2,289 | (0.8) | 1,436 | (2.2) |
|  | Schizophrenia | 5,805 | (2.0) | 3,786 | (1.3) | 2,019 | (0.7) | 1,872 | (2.8) |
|  | Bipolar disorder | 1,160 | (0.4) | 775 | (0.3) | 385 | (0.1) | 481 | (0.7) |
|  | Personality disorder | 673 | (0.2) | 442 | (0.2) | 231 | (0.1) | 226 | (0.3) |
|  | Intellectual disability | 486 | (0.2) | 360 | (0.1) | 126 | (0.0) | 94 | (0.1) |
|  | OCD | 300 | (0.1) | 215 | (0.1) | 85 | (0.0) | 82 | (0.1) |
|  | Eating disorder | 37 | (0.0) | 28 | (0.0) | 9 | (0.0) | 11 | (0.0) |
| **Epilepsy** |  | 4,306 | (1.5) | 3,152 | (1.1) | 1,154 | (0.4) | 864 | (1.3) |
| **Diabetes** |  | 70,817 | (23.9) | 37,439 | (12.6) | 33,378 | (11.3) | 14,982 | (22.7) |
| **Cardiovascular Disease** | TOTAL | 125,531 | (42.4) | 65,915 | (22.3) | 59,616 | (20.1) | 26,788 | (40.7) |
|  | Hyperlipidaemia | 61,677 | (20.8) | 33,250 | (11.2) | 28,427 | (9.6) | 12,467 | (18.9) |
|  | Cerebrovascular disease | 54,863 | (18.5) | 29,512 | (10.0) | 25,351 | (8.6) | 11,404 | (17.3) |
|  | ASCVD | 44,984 | (15.2) | 22,577 | (7.6) | 22,407 | (7.6) | 10.056 | (15.3) |
|  | Congestive Heart Failure | 29,743 | (10.1) | 14,030 | (4.7) | 15,713 | (5.3) | 6,147 | (9.3) |
|  | Myocardial infarction | 16,209 | (5.5) | 8,065 | (2.7) | 8,144 | (2.8) | 3,179 | (4.8) |
|  | Peripheral vascular disease | 6,052 | (2.0) | 3,121 | (1.1) | 2,931 | (1.0) | 1,198 | (1.8) |
| **Cancer** | TOTAL | 58,093 | (19.6) | 28,667 | (9.7) | 29,426 | (9.9) | 13,086 | (19.9) |
|  | Colorectum | 12,047 | (4.1) | 5,942 | (2.0) | 6,105 | (2.1) | 2,458 | (3.7) |
|  | Lung | 11,268 | (3.8) | 5,523 | (1.9) | 5,745 | (1.9) | 3,079 | (4.7) |
|  | Breast | 6,952 | (2.4) | 2,992 | (1.0) | 3,960 | (1.3) | 1,621 | (2.5) |
|  | Male genital organ | 5,330 | (1.8) | 2,497 | (0.8) | 2,833 | (1.0) | 1,115 | (1.7) |
|  | Female genital organ | 3,660 | (1.2) | 1,743 | (0.6) | 1,917 | (0.7) | 869 | (1.3) |
|  | Lip/Oral/Pharynx | 3,152 | (1.1) | 1,746 | (0.6) | 1,406 | (0.5) | 695 | (1.1) |
|  | Liver | 2,968 | (1.0) | 1,521 | (0.5) | 1,447 | (0.5) | 581 | (0.9) |
|  | Bladder | 2,543 | (0.9) | 1,309 | (0.4) | 1,234 | (0.4) | 518 | (0.8) |
|  | Stomach | 2,307 | (0.8) | 1,108 | (0.4) | 1,199 | (0.4) | 462 | (0.7) |
|  | Bone/skin/mesothelial | 2,281 | (0.8) | 1,168 | (0.4) | 1,113 | (0.4) | 501 | (0.8) |
|  | Kidney | 1,365 | (0.5) | 677 | (0.2) | 688 | (0.2) | 337 | (0.5) |
|  | Pancreas | 1,239 | (0.4) | 540 | (0.2) | 699 | (0.2) | 239 | (0.4) |
|  | Lymphoid | 675 | (0.2) | 291 | (0.1) | 384 | (0.1) | 117 | (0.2) |
|  | Brain/CNS | 240 | (0.1) | 163 | (0.1) | 77 | (0.0) | 56 | (0.1) |

*BZD: benzodiazepine, SUD: substance use disorder, OCD: obsessive-compulsive disorder, ADHD: attention deficit hyperactivity disorder, ASCVD: atherosclerotic cardiovascular disease, CNS: central nervous system.

**Note: The BZD, Z-drug, and BZD&Z-drug groups are not mutually exclusive; a patient may be included in more than one group.

eTable 5. Results of joinpoint analysis of the prevalence of BZD and Z-drug prescribing by sex and age groups.

|  | **AAPC [95% C.I.]** | | **P-value** | **APC [95% C.I.]** | **P-value** | | **Segment** |
| --- | --- | --- | --- | --- | --- | --- | --- |
| **Overall Prevalence** | | | | | | | |
| **By Sex** | | | | | | | |
| Male | 3.70 [3.25, 4.05] | | <0.001 | 4.93 [4.58, 5.49] | <0.001 | | 2014-2021 |
|  |  | |  | -0.51 [-2.73, 1.87] | 0.53 | | 2021-2023 |
| Female | 3.25 [2.97, 3.56] | | <0.001 | 3.90 [3.61, 4.48] | <0.001 | | 2014-2021 |
|  |  | |  | 1.02 [-0.38, 2.80] | 0.15 | | 2021-2023 |
| **By Age** | | | | | | | |
| 18-25 | 9.43 [8.36, 10.51] | | <0.001 |  |  | | 2014-2023 |
| 26-49 | 0.87 [0.45, 1.27] | | <0.001 |  |  | | 2014-2023 |
| 50-64 | 2.04 [1.75, 2.29] | | <0.001 | 2.95 [2.69, 3.35] | <0.001 | | 2014-2021 |
|  |  | |  | -1.11 [-2.57, 0.70] | 0.21 | | 2021-2023 |
| 65+ | 2.4 [2.21, 2.61] | | <0.001 | 4.38 [3.93, 5.37] | <0.001 | | 2014-2018 |
|  |  | |  | 2.95 [2.41, 3.78] | <0.001 | | 2018-2021 |
|  |  | |  | -2.24 [-3.20, -1.08] | <0.001 | | 2021-2023 |
| **BZD Prevalence** | | | | | | | |
| **By Sex** | | | | | | | |
| Male | | 3.98 [3.56, 4.33] | <0.001 | 5.42 [5.04, 5.95] | <0.001 | 2014-2021 | |
|  | |  |  | -0.91 [-2.93, 1.69] | 0.33 | 2021-2023 | |
| Female | | 3.11 [2.72, 3.56] | <0.001 | 3.77 [3.38, 5.07] | <0.001 | 2014-2021 | |
|  | |  |  | 0.84 [-1.11, 2.92] | 0.29 | 2021-2023 | |
| **By Age** | | | | | | | |
| 18-25 | 10.25 [9.01, 11.46] | | <0.001 |  |  | |  |
| 26-49 | 1.2 [0.67, 1.71] | | <0.001 |  |  | |  |
| 50-64 | 1.85 [1.41, 2.24] | | <0.001 | 2.83 [2.46, 3.50] | <0.001 | | 2014-2021 |
|  |  | |  | -1.52 [-3.72, 0.80] | 0.19 | | 2021-2023 |
| 65+ | 2.91 [2.48, 3.28] | | <0.001 | 4.67 [4.26, 5.20] | <0.001 | | 2014-2021 |
|  |  | |  | -3.01 [-5.10, -0.11] | 0.036 | | 2021-2023 |
| **Z-drug Prevalence** | | | | | | | |
| **By Sex** |  | |  |  |  | |  |
| Male | 3.09 [2.94, 3.22] | | <0.001 | 4.24 [4.06, 4.42] | <0.001 | | 2014-2021 |
|  |  | |  | -0.85 [-1.58, -0.17] | 0.027 | | 2021-2023 |
| Female | 3.35 [3.18, 3.49] | | <0.001 | 3.57 [2.79, 4.08] | <0.001 | | 2014-2017 |
|  |  | |  | 4.61 [4.28, 5.11] | <0.001 | | 2017-2021 |
|  |  | |  | 0.55 [-0.23, 0.35 | 0.18 | | 2021-2023 |
| **By Age** |  | |  |  |  | |  |
| 18-25 | 5.72 [5.11, 6.28] | | <0.001 | 2.64 [-0.94, 4.70] | 0.098 | | 2014-2017 |
|  |  | |  | 11.91 [10.53, 14.38] | <0.001 | | 2017-2021 |
|  |  | |  | -1.38 [-4.11, 2.45] | 0.29 | | 2021-2023 |
| 26-49 | -0.58 [-0.92, -0.28] | | <0.001 | -1.23 [-2.84, -0.24] | 0.011 | | 2014-2017 |
|  |  | |  | 0.95 [0.40, 2.08] | 0.004 | | 2017-2021 |
|  |  | |  | -2.61 [-4.27, -1.02] | <0.001 | | 2021-2023 |
| 50-64 | 2.43 [2.29, 2.59] | | <0.001 | 3.63 [3.29, 4.38] | <0.001 | | 2014-2018 |
|  |  | |  | 2.92 [2.53, 3.42] | <0.001 | | 2018-2021 |
|  |  | |  | -0.62 [-1.40, 0.38] | 0.11 | | 2021-2023 |
| 65+ | 2.12 [1.78, 2.48] | | <0.001 | 4.07 [3.06, 5.97] | <0.001 | | 2014-2018 |
|  |  | |  | 2.57 [1.93, 4.14] | <0.001 | | 2018-2021 |
|  |  | |  | -2.29 [-4.06, 0.37] | 0.018 | | 2021-2023 |

*AAPC: average annual percentage change during the whole study period, APC: annual percentage change during the corresponding segment; BZD: benzodiazepine

eTable 6. Result of joinpoint analysis on prevalence and incidence of seven highly prevalent BZDs and Z-drugs.

|  | **Prevalence** | | | | | **Incidence** | | | | |
| --- | --- | --- | --- | --- | --- | --- | --- | --- | --- | --- |
|  | **AAPC [95% C.I.]** | **P-value** | **APC [95% C.I.]** | **P-value** | **Segment** | **AAPC [95% C.I.]** | **P-value** | **APC [95% C.I.]** | **P-value** | **Segment** |
| **Alprazolam** | 4.32 [3.83, 4.70] | <0.001 | 3.97 [1.92, 4.95] | <0.001 | 2014-2018 | 2.5 [1.60, 3.35] | <0.001 |  |  | 2014-2023 |
|  |  |  | 6.25 [5.02, 7.43] | <0.001 | 2018-2021 |  |  |  |  |  |
|  |  |  | 2.18 [-0.07, 4.22] | 0.056 | 2021-2023 |  |  |  |  |  |
| **Clonazepam** | 4.51 [4.38, 4.61] | <0.001 | 5.11 [4.99, 5.26] | 0.011 | 2014-2021 | 0.85 [-0.05, 1.75] | 0.066 |  |  | 2014-2023 |
|  |  |  | 2.43 [1.79, 3.42] | <0.001 | 2021-2023 |  |  |  |  |  |
| **Diazepam** | -0.87 [-1.59, 0.001] | 0.051 | -0.23 [-1.74, 3.66] | 0.76 | 2014-2021 | -2.18 [-3.18, -1.20] | <0.001 |  |  | 2014-2023 |
|  |  |  | -3.1 [-6.66, 0.01] | 0.054 | 2021-2023 |  |  |  |  |  |
| **Lorazepam** | 4.43 [4.12, 4.82] | <0.001 | 5.22 [4.85, 6.05] | <0.001 | 2014-2019 | 2.1 [0.86, 3.63] | <0.001 | 3.44 [1.06, 9.94] | 0.021 | 2014-2021 |
|  |  |  | 1.72 [0.21, 3.94] | 0.021 | 2019-2023 |  |  | -2.44 [-8.51, 3.38] | 0.52 | 2021-2023 |
| **Midazolam** | 14.41 [12.88, 16.50] | <0.001 | 25.63 [21.45, 31.01] | <0.001 | 2014-2019 | 15.35 [13.17, 17.48] | <0.001 | 27.24 [22.28, 33.73] | <0.001 | 2014-2019 |
|  |  |  | 2.38 [-3.51, 7.20] | 0.33 | 2019-2023 |  |  | 2.04 [-5.35, 7.51] | 0.47 | 2019-2023 |
| **Zolpidem** | 3.56 [3.42, 3.69] | <0.001 | 4.82 [4.12, 5.32] | <0.001 | 2014-2016 | -0.43 [-1.08, 0.24] | 0.19 | 0.99 [-0.01, 3.85] | 0.054 | 2014-2019 |
|  |  |  | 3.87 [3.46, 4.17] | <0.001 | 2016-2021 |  |  | -2.17 [-5.33, -0.84] | 0.001 | 2019-2023 |
|  |  |  | 1.56 [1.01, 2.32] | <0.001 | 2021-2023 |  |  |  |  |  |
| **Zopiclone** | 2.98 [2.87, 3.09] | <0.001 | 3.54 [2.94, 3.91] | <0.001 | 2014-2017 | -0.58 [-1.34, 0.19] | 0.12 | 0.99 [0.32, 2.46] | 0.005 | 2014-2021 |
|  |  |  | 4.46 [4.21, 4.83] | <0.001 | 2017-2021 |  |  | -5.87 [-9.54, -1.71] | <0.001 | 2021-2023 |
|  |  |  | -0.72 [-1.26, -0.25] | <0.001 | 2021-2023 |  |  |  |  |  |

*AAPC: average annual percentage change during the whole study period, APC: annual percentage change during the corresponding segment, BZD: benzodiazepines

eTable 7. Results of joinpoint analysis of incidence rate of BZD and Z-drug prescribing by sex and age groups.

|  | **AAPC [95% C.I.]** | **P-value** | **APC [95% C.I.]** | **P-value** | **Segment** |
| --- | --- | --- | --- | --- | --- |
| **Overall Incidence rate** | | | | | |
| **By Sex** |  |  |  |  |  |
| Male | 2.97 [2.37, 3.59] |  | 5.29 [4.07, 8.03] | <0.001 | 2014-2019 |
|  |  |  | 0.13 [-3.12, 1.81] | 0.92 | 2019-2023 |
| Female | 0.74 [0.28, 1.32] |  | 1.91 [1.37, 3.08] | <0.001 | 2014-2021 |
|  |  |  | -3.27 [-5.49, 0.03] | 0.051 | 2021-2023 |
| **By Age** |  |  |  |  |  |
| 18-25 | 7.56 [6.19, 8.89] | <0.001 |  |  | 2014-2023 |
| 26-49 | -0.82 [-1.37, -0.12] | 0.022 | 0.14 [-0.56, 2.40] | 0.46 | 2014-2021 |
|  |  |  | -4.1 [-6.69, -0.84] | 0.007 | 2021-2023 |
| 50-64 | 0.04 [-0.76, 1.00] | 0.73 | 1.58 [0.84, 3.69] | 0.002 | 2014-2021 |
|  |  |  | -5.14 [-9.01, -0.65] | 0.008 | 2021-2023 |
| 65+ | 0.67 [0.07, 1.27] | 0.028 | 3.55 [2.25, 5.51] | <0.001 | 2014-2019 |
|  |  |  | -2.81 [-5.55, -1.18] | <0.001 | 2019-2023 |
| **BZD Incidence rate** | | | | | |
| **By Sex** |  |  |  |  |  |
| Male | 3.95 [3.13, 4.73] | <0.001 | 6.55 [5.36, 8.28] | <0.001 | 2014-2020 |
|  |  |  | -1.08 [-5.83, 1.77] | 0.46 | 2020-2023 |
| Female | 1.25 [0.40, 2.18] | 0.003 | 2.69 [1.93, 4.99] | 0.003 | 2014-2021 |
|  |  |  | -3.63 [-7.73, 0.73] | 0.13 | 2021-2023 |
| **By Age** |  |  |  |  |  |
| 18-25 | 8.76 [7.28, 10.15] | <0.001 |  | <0.001 | 2014-2023 |
| 26-49 | -0.5 [-1.25, 0.41] | 0.3 | 0.55 [-0.54, 3.91] | 0.14 | 2014-2021 |
|  |  |  | -4.07 [-7.63, -0.21] | 0.039 | 2021-2023 |
| 50-64 | 0.28 [-0.62, 1.26] | 0.41 | 2.16 [1.33, 4.06] | <0.001 | 2014-2021 |
|  |  |  | -6.03 [-10.27, -0.86] | 0.01 | 2021-2023 |
| 65+ | 2.61 [1.27, 3.74] | <0.001 | 5.95 [4.38, 8.49] | <0.001 | 2014-2020 |
|  |  |  | -3.77 [-11.11, -0.06] | 0.047 | 2020-2023 |
| **Z-drug Incidence rate** | | | | | |
| **By Sex** |  |  |  |  |  |
| Male | 0.07 [-0.36, 0.48] | 0.73 | 2.38 [1.43, 3.67] | <0.001 | 2014-2019 |
|  |  |  | -2.74 [-4.62, -1.56] | <0.001 | 2019-2023 |
| Female | -0.54 [-1.45, 0.51] | 0.22 | 0.71 [-0.09, 4.60] | 0.073 | 2014-2021 |
|  |  |  | -4.80 [-9.19, -0.66] | 0.024 | 2021-2023 |
| **By Age** |  |  |  |  |  |
| 18-25 | 2.62 [1.95, 3.21] | <0.001 | 2.41 [-0.75, 4.54] | 0.13 | 2014-2017 |
|  |  |  | 7.62 [6.27, 9.97] | <0.001 | 2017-2021 |
|  |  |  | -6.4 [-9.44, -2.10] | <0.001 | 2021-2023 |
| 26-49 | -3.2 [-3.88, -2.42] | <0.001 | -2.13 [-2.75, 0.13] | 0.053 | 2014-2021 |
|  |  |  | -6.86 [-10.13, -3.41] | <0.001 | 2021-2023 |
| 50-64 | -1.08 [-1.93, -0.32] | 0.012 | 0.85 [-0.08, 2.58] | 0.076 | 2014-2020 |
|  |  |  | -4.82 [-9.63, -2.44] | <0.001 | 2020-2023 |
| 65+ | -1.73 [-2.50, -1.00] | <0.001 | 0.98 [-0.40, 3.32] | 0.16 | 2014-2019 |
|  |  | <0.001 | -5.03 [-8.06, -3.30] | <0.001 | 2019-2023 |

*AAPC: average annual percentage change during the whole study period; APC: annual percentage change during the corresponding segment; BZD: benzodiazepine

eTable 8**.** The proportion of patients with psychiatric diagnoses made within 180 days before or after the incident BZD or Z-drug prescriptions.

|  |  | | **2014** | **2015** | **2016** | **2017** | **2018** | **2019** | **2020** | **2021** | **2022** | **2023** |
| --- | --- | --- | --- | --- | --- | --- | --- | --- | --- | --- | --- | --- |
| *Overall, % | |  | 45.6 | 43.9 | 42.2 | 39.4 | 38.4 | 35.4 | 36.3 | 36.5 | 36.1 | 35.3 |
| Age group: | | 18-25 | 82.2 | 78.7 | 80.3 | 75.4 | 73.9 | 71.3 | 77.2 | 79.2 | 76.1 | 76.5 |
|  | | Over 65 | 34.6 | 32.6 | 31.1 | 29.1 | 28.0 | 25.9 | 26.5 | 27.0 | 26.0 | 25.3 |
| Depression | |  | 15.8 | 15.0 | 14.0 | 13.2 | 12.7 | 11.7 | 12.4 | 12.0 | 11.5 | 11.0 |
|  |  | 18-25 | 25.2 | 23.5 | 25.6 | 27.2 | 26.9 | 26.7 | 32.0 | 33.0 | 29.8 | 30.8 |
|  |  | Over 65 | 7.1 | 6.8 | 6.3 | 5.8 | 5.4 | 5.0 | 5.9 | 5.7 | 5.7 | 5.5 |
| Dementia | |  | 6.8 | 6.4 | 6.4 | 6.2 | 6.0 | 5.4 | 5.3 | 5.7 | 5.4 | 5.0 |
|  |  | 18-25 |  |  |  | 0.0 |  |  |  | 0.0 |  |  |
|  |  | Over 65 | 16.7 | 15.4 | 14.7 | 13.7 | 13.4 | 11.3 | 11.0 | 11.9 | 10.8 | 9.8 |
| Schizophrenia | |  | 5.1 | 4.9 | 4.8 | 4.2 | 4.1 | 3.4 | 3.4 | 3.2 | 3.2 | 3.1 |
|  |  | 18-25 | 13.7 | 11.2 | 10.6 | 9.4 | 8.8 | 8.7 | 6.7 | 6.3 | 6.8 | 6.3 |
|  |  | Over 65 | 1.4 | 1.3 | 1.3 | 1.2 | 1.2 | 1.1 | 1.1 | 1.1 | 1.0 | 1.0 |
| Other psychosis | |  | 4.0 | 4.0 | 3.7 | 3.1 | 3.3 | 2.8 | 2.9 | 3.1 | 3.4 | 3.6 |
|  |  | 18-25 | 13.8 | 13.6 | 13.3 | 10.4 | 10.2 | 10.1 | 9.5 | 9.0 | 9.8 | 9.4 |
|  |  | Over 65 | 1.8 | 1.9 | 1.8 | 1.6 | 1.6 | 1.5 | 1.4 | 1.6 | 1.8 | 2.0 |
| AD | |  | 3.6 | 3.4 | 3.3 | 3.2 | 3.1 | 3.0 | 3.4 | 3.6 | 3.4 | 3.4 |
|  |  | 18-25 | 4.8 | 4.2 | 5.3 | 5.4 | 5.0 | 4.5 | 5.4 | 7.2 | 6.9 | 6.3 |
|  |  | Over 65 | 1.9 | 1.8 | 1.5 | 1.6 | 1.5 | 1.6 | 1.9 | 1.9 | 1.8 | 1.9 |
| SUD | |  | 3.0 | 3.1 | 3.1 | 3.0 | 3.0 | 3.2 | 3.2 | 3.0 | 2.8 | 2.8 |
|  |  | 18-25 | 5.7 | 6.2 | 5.8 | 5.9 | 4.7 | 5.0 | 5.1 | 4.7 | 4.2 | 4.2 |
|  |  | Over 65 | 0.9 | 0.9 | 1.0 | 0.9 | 0.9 | 1.6 | 1.3 | 1.0 | 1.0 | 0.9 |
| SDD | |  | 2.8 | 2.8 | 2.8 | 2.7 | 2.5 | 2.6 | 2.5 | 2.8 | 3.1 | 3.2 |
|  |  | 18-25 | 2.5 | 2.8 | 2.5 | 2.5 | 2.6 | 2.5 | 2.4 | 2.6 | 2.3 | 3.0 |
|  |  | Over 65 | 1.9 | 2.3 | 2.2 | 2.2 | 2.0 | 2.0 | 2.1 | 2.1 | 2.3 | 2.5 |
| BD | |  | 1.5 | 1.4 | 1.3 | 1.3 | 1.3 | 1.1 | 1.1 | 1.1 | 1.1 | 1.2 |
|  |  | 18-25 | 5.3 | 5.4 | 5.9 | 4.6 | 4.9 | 4.6 | 5.6 | 5.7 | 6.1 | 6.3 |
|  |  | Over 65 | 0.3 | 0.3 | 0.3 | 0.3 | 0.3 | 0.2 | 0.2 | 0.3 | 0.2 | 0.3 |
| PD | |  | 1.0 | 0.9 | 0.9 | 0.8 | 0.8 | 0.8 | 0.8 | 0.7 | 0.6 | 0.7 |
|  |  | 18-25 |  |  |  |  |  | 0.0 | 0.0 |  |  |  |
|  |  | Over 65 | 2.3 | 1.8 | 1.8 | 1.7 | 1.6 | 1.5 | 1.4 | 1.2 | 1.1 | 1.1 |
| ID | |  | 0.9 | 0.9 | 0.9 | 0.8 | 0.8 | 0.7 | 0.7 | 0.6 | 0.7 | 0.7 |
|  |  | 18-25 | 5.5 | 5.9 | 5.3 | 5.0 | 5.5 | 4.8 | 4.7 | 3.8 | 4.5 | 4.4 |
|  |  | Over 65 | 0.1 | 0.1 | 0.1 | 0.1 | 0.1 | 0.1 | 0.1 | 0.1 | 0.1 | 0.1 |
| Personality disorder | |  | 0.7 | 0.7 | 0.7 | 0.5 | 0.5 | 0.4 | 0.4 | 0.5 | 0.4 | 0.4 |
|  |  | 18-25 | 3.2 | 3.3 | 2.9 | 2.4 | 2.2 | 1.6 | 2.4 | 3.2 | 2.2 | 2.3 |
|  |  | Over 65 | 0.2 | 0.2 | 0.1 | 0.1 | 0.1 | 0.1 | 0.1 | 0.1 | 0.1 | 0.1 |
| OCD | |  | 0.3 | 0.3 | 0.3 | 0.3 | 0.2 | 0.2 | 0.2 | 0.2 | 0.2 | 0.2 |
|  |  | 18-25 | 1.7 | 1.2 | 2.0 | 1.7 | 1.5 | 1.3 | 1.4 | 1.6 | 1.0 | 1.3 |
|  |  | Over 65 | 0.0 | 0.0 | 0.1 | 0.0 | 0.0 | 0.0 | 0.0 | 0.0 | 0.0 | 0.0 |
| ADHD | |  | 0.0 | 0.0 | 0.0 | 0.1 | 0.1 | 0.1 | 0.1 | 0.1 | 0.1 | 0.1 |
|  |  | 18-25 | 0.2 | 0.5 | 0.6 | 0.6 | 0.8 | 0.8 | 1.4 | 1.3 | 2.1 | 1.8 |
|  |  | Over 65 |  |  |  |  |  |  |  |  |  |  |
| More than one diagnosis | |  | 5.9 | 6.0 | 5.6 | 4.8 | 4.6 | 4.1 | 4.4 | 4.4 | 4.6 | 4.6 |
|  |  | 18-25 | 17.0 | 16.1 | 16.2 | 14.4 | 12.1 | 12.1 | 13.3 | 13.6 | 13.1 | 13.6 |
|  |  | Over 65 | 3.4 | 3.3 | 3.0 | 2.6 | 2.5 | 2.3 | 2.4 | 2.3 | 2.4 | 2.5 |

*AD: anxiety disorder; SUD: substance use disorder; SDD: sleep disorder/disturbance; BD: bipolar disorder; PD: Parkinson’s disease; ID: intellectual disability; OCD: obsessive compulsive disorder; ADHD: attention deficit hyperactivity disorder

**Note: the proportion was calculated by dividing the number of patients with corresponding psychiatric diseases by the total number of patients with incident BZD or Z-drug prescriptions in percentage.

eTable 9. Prescribing duration of BZDs and Z-drugs prescriptions started between January 1^st^, 2014 and December 31^st^, 2022.

|  | **BZD** | | **Z-drug** | |
| --- | --- | --- | --- | --- |
| Total Prescription No. | 723,851 | | 618,176 | |
| Duration | Prescription No. | Prop. (%) | Prescription No. | Prop. (%) |
| 1-30 days | 398,628 | 55.1 | 232,200 | 37.6 |
| 31-90 days | 89,472 | 12.4 | 98,362 | 15.9 |
| 91-180 days | 65,730 | 9.1 | 78,143 | 12.6 |
| 181-365 days | 49,153 | 6.8 | 58,961 | 9.5 |
| ≥ 366 days | 120,868 | 16.7 | 150,510 | 24.3 |

*BZD: Benzodiazepine, Prop.: Proportion (Patient number of respective duration/total patient number *100)

**Note: prescribing duration was evaluated after merging any sequential, overlapping, or gap-separated prescriptions with gaps of less than 30 days.

eTable 10. Results of joinpoint regression analysis on the annual prevalence of patients with long-term BZD and Z-drug prescriptions, specifically by sex and age groups.

|  | **AAPC [95% C.I.]** | | **APC [95% C.I.]** | | **P-value** | **Segment** |
| --- | --- | --- | --- | --- | --- | --- |
| **Long-term BZD** | | | | | | |
| > 90 days | 1.77 | [0.92, 2.65] |  |  | <0.001 | 2014-2022 |
| **By Sex** |  |  |  |  |  |  |
| Male | 1.34 | [0.28, 2.40] |  |  | 0.016 | 2014-2022 |
| Female | 1.97 | [1.25, 2.71] |  |  | <0.001 | 2014-2022 |
| **By Age** |  |  |  |  |  |  |
| 18-25 | 13.43 | [11.98, 14.62] | 10.45 | [4.82, 12.60] | <0.001 | 2014-2019 |
|  |  |  | 18.58 | [14.00, 25.44] | <0.001 | 2019-2022 |
| 26-49 | -0.05 | [-0.62, 0.54] |  |  | 0.85 | 2014-2022 |
| 50-65 | 0.66 | [-0.58, 1.91] |  |  | 0.27 | 2014-2022 |
| 65+ | 0.46 | [-1.09, 2.06] |  |  | 0.53 | 2014-2022 |
| **Long-term Z-drug** | | | | | | |
| > 90 days | 2.29 | [1.92, 2.71] | 3.26 | [2.84, 4.07] | <0.001 | 2014-2020 |
|  |  |  | -0.55 | [-2.19, 1.56] | 0.70 | 2020-2022 |
| **By Sex** |  |  |  |  |  |  |
| Male | 1.88 | [1.16, 2.73] | 3.23 | [2.52, 5.51] |  | 2014-2020 |
|  |  |  | -2.08 | [-5.19, 1.40] | 0.30 | 2020-2022 |
| Female | 2.48 | [1.93, 3.18] | 3.21 | [2.61, 5.66] |  | 2014-2020 |
|  |  |  | 0.32 | [-2.20, 2.56] | 0.52 | 2020-2022 |
| **By Age** |  |  |  |  |  |  |
| 18-25 | 12.88 | [7.85, 18.24] |  |  | <0.001 | 2014-2022 |
| 26-49 | -0.2 | [-0.51, 0.08] | -1.74 | [-3.34, -0.95] | <0.001 | 2014-2017 |
|  |  |  | 2.93 | [2.00, 3.69] | <0.001 | 2017-2020 |
|  |  |  | -2.49 | [-3.72, -1.29] | <0.001 | 2020-2022 |
| 50-64 | 1.85 | [0.58, 3.46] | 3.25 | [1.23, 9.33] | 0.016 | 2014-2020 |
|  |  |  | -2.24 | [-7.80, 2.88] | 0.39 | 2020-2022 |
| 65+ | -0.39 | [-0.90, 0.22] | 1.16 | [0.46, 2.26] | <0.001 | 2014-2020 |
|  |  |  | -4.79 | [-6.95, -1.72] | <0.001 | 2020-2022 |

*BZD: Benzodiazepine; AAPC: average annual percentage change during the whole study period; APC: annual percentage change during the corresponding segment

eTable 11. Results of joinpoint regression analysis on the annual prevalence of patients with BZD and Z-drug prescriptions based on specific treatment duration.

| **BZD** | **AAPC [95% C.I.]** | **P-value** | **APC [95% C.I.]** | **P-value** | **Segment** |
| --- | --- | --- | --- | --- | --- |
| 1-30 days | 5.09 [3.59, 6.43] | <0.001 | 7.40 [5.62, 12.67] | <0.001 | 2014-2019 |
|  |  |  | 1.36 [-4.16, 4.76] | 0.51 | 2019-2022 |
| 31-90 days | 0.48 [-0.41, 1.39] | 0.28 |  |  | 2014-2022 |
| 91-180 days | -2.37 [-3.93, -0.78] | <0.001 |  |  | 2014-2022 |
| 181-365 days | 2.55 [1.57, 3.50] | <0.001 |  |  | 2014-2022 |
| > 365 days | 3.70 [2.91, 4.49] | <0.001 |  |  | 2014-2022 |
| **Z-drug** |  |  |  |  |  |
| 1-30 days | -0.77 [-2.02, 0.51] | 0.21 |  |  | 2014-2022 |
| 31-90 days | 1.46 [0.11, 3.06] | 0.037 | 3.53 [2.36, 8.50] | 0.007 | 2014-2020 |
|  |  |  | -4.52 [-10.47, 0.99] | 0.13 | 2020-2022 |
| 91-180 days | -1.67 [-3.33, 0.02] | 0.054 |  |  | 2014-2022 |
| 181-365 days | 2.94 [2.17, 3.88] | <0.001 | 3.9 [2.74, 7.20] | 0.054 | 2014-2020 |
|  |  |  | 0.11 [-3.24, 3.31] | 0.67 | 2020-2022 |
| > 365 days | 4.57 [3.95, 5.26] | <0.001 | 5.90 [5.26, 7.42] | <0.001 | 2014-2020 |
|  |  |  | 0.66 [-2.05, 3.89] | 0.48 | 2020-2022 |

*BZD: Benzodiazepine; AAPC: average annual percentage change during the whole study period; APC: annual percentage change during the corresponding segment.

eTable 12. Results of the joinpoint analysis of the prevalence and incidence of patients with BZD and Z-drug prescribing before COVID-19 and after COVID-19.

|  | **Pre-COVID-19** | | **Post-COVID-19** | |
| --- | --- | --- | --- | --- |
|  | **AAPC [95% CI]** | **P-value** | **AAPC [95% CI]** | **P-value** |
| **Prevalence** |  |  |  |  |
| Overall | 4.03 [4.01, 4.58] | <0.001 | 1.84 [-1.34, 5.10] | 0.26 |
| BZD | 4.43 [4.09, 4.77 | <0.001 | 1.74 [-1.73, 5.27] | 0.31 |
| Z-drug | 4.21 [3.88, 4.55] | <0.001 | 1.58 [-1.18, 4.39] | 0.26 |
| **Incidence** |  |  |  |  |
| Overall | 3.29 [2.63, 3.96] | <0.001 | -0.88 [-5.72, 4.08] | 0.68 |
| BZD | 4.47 [3.77, 5.16] | <0.001 | -0.88 [-6.27, 4.73] | 0.71 |
| Z-drug | 1.62 [1.11, 2.13] | <0.001 | -2.88 [-7.20, 1.63] | 0.21 |

*AAPC: average annual percentage change during the whole study period; BZD: benzodiazepine

**Note: pre-COVID-19 period is from 2014 to 2019, and post-COVID-19 is from 2020 to 2023. No significant trend change points were detected in this analysis, which means AAPC equates APC.

**eFigure 1. Annual prevalence and incidence of seven individual drugs that are highly prevalent.** a) Prevalence of individual drugs b) Incidence of individual drugs.

Note: the red points represent significant changes in trends as identified by the joinpoint analysis. * indicates that annual percentage change (APC) is significantly different from zero at the alpha=0.05 level.

**eFigure 2. Subgroup analysis of the annual prevalence of patients by the duration of prescription periods**. a) Prevalence by prescribing durations: BZD b) Prevalence by prescribing durations: Z-drug.

Note: the red points represent significant changes in trends as identified by the joinpoint analysis. * indicates that annual percentage change (APC) is significantly different from zero at the alpha=0.05 level.

**eFigure 3. Sensitivity analysis of the annual prevalence of patients with different prescribing durations as long-term.** Periods exceeding 180 days and 365 days were evaluated for a) patients with BZD prescription and b) patients with Z-drug prescription.

Note: the red points represent significant changes in trends as identified by the joinpoint analysis. * indicates that annual percentage change (APC) is significantly different from zero at the alpha=0.05 level.

**Reference**

1. Greenblatt DJ, Shader RI, Divoll M, Harmatz JS. Benzodiazepines: a summary of pharmacokinetic properties. *Br J Clin Pharmacol.* 1981;11 Suppl 1(Suppl 1):11S-16S.

2. Procyshyn RM, Bezchlibnyk-Butler, K. Z., & Jeffries, J. J. *Clinical handbook of psychotropic drugs.* 21st ed. ed: Hogrefe Publishing; 2015.
